# Impacts of Fruit Frosting Coverage on Postharvest Softening of Prunes under Vibration Stress

**DOI:** 10.3390/foods13193197

**Published:** 2024-10-08

**Authors:** Wanting Chen, Kuanbo Cui, Lili Jin, Menghan Bai, Ohaer Pazilijiang, Rui Tian, Junjie Ma

**Affiliations:** 1 College of Food Science and Pharmacy, Xinjiang Agricultural University, Urumqi 830052, China; m17884806613@163.com (W.C.); jl826850654@163.com (L.J.); baimenghan0215@163.com (M.B.); 18963807550@163.com (O.P.); 15299831248@163.com (J.M.); 2 Agricultural Mechanization, Xinjiang Academy of Agricultural Sciences, Urumqi 830091, China; 3 College of Chemistry and Chemical Engineering, Xinjiang Agricultural University, Urumqi 830052, China; tianrui2024xjnd@126.com

**Keywords:** prunes, fruit frosting, vibration stress, cell wall metabolism, postharvest quality

## Abstract

The surface of prune fruit has a thick layer of frosting, which is easily damaged and lost during prunes harvest or postharvest handling, and there is no clear information on the effect of prune surface frost on postharvest storage quality. To investigate the effect of fruit frosting on the softening of prune fruits during storage under vibration stress, prunes were divided into three grades according to fruit frosting in this study and were vibrated for 8 h at a frequency of 5 Hz at 4 °C; then, samples were selected once every 8 d. The results showed that the heavy fruit frosting (HFF) group maintained higher hardness (21.47%), L* (20.85%), and total soluble solids (12.79%) levels at the end of storage and inhibited cell wall-modifying enzyme activities (polygalacturonase, pectin methylesterase, glycosidase, *β*-glucosidase, and cellulase) compared to frosting-less fruit (FF) group. This group also showed improved expression of key cell wall-modification genes (*ADPG2*, *PME31*, *CESA1*, *BGAL3*, *XTH33*, *BGLU41*) as well as chelate-soluble pectin (72.11%), Na_2_CO_3_-soluble pectin (42.83%), and cellulose (36.89%) solubilization and maintained lower water-soluble pectin (34.23%). Microscopic observations showed that the fruit frosting could delay the dissolution of pectin components and protect the cell wall structure. In summary, fruit frosting can effectively inhibit fruit softening and maintain fruit quality.

## 1. Introduction

Prune, a member of the Rosaceae family, is widely planted in temperate and continental climate zones globally [1]. The sweet and sour tastes and rich nutritional value of prunes make them popular with consumers [2]. With the increasing demand and unique climatic advantages found in Xinjiang, Kashgar, it is currently the largest prune production, export, and processing base in China. However, prunes are a respiratory change type of fruit, meaning that after harvest, if the storage conditions are not appropriate, there will be water, aroma, and nutrients loss. Also, the speed of fruit softening will be accelerated, mainly manifesting as a decline in hardness, the collapse of the internal structure of the fruit, the decomposition of sugars, etc. All of this means that in the process of storage, transport, and marketing, there could be huge losses to fruit growers [3], and maintaining fruit quality is crucial. Therefore, we explored storage methods for pruned fruits.

Prune skin is covered with white fruit frosting, a fruit wax it secrets that adheres to the outermost layer of the fruit, as in blueberries [4], plums [5], and grapes [6]. The epidermal wax layer of fruits is closely related to their postharvest physiological quality. For example, blueberry epidermal wax layer removal exacerbates quality cracking during storage [7]; cantaloupe dewaxing leads to increased accumulation of reactive oxygen species and decreased antioxidant system defenses, which ultimately leads to fruit senescence [8]; and grape epidermal wax removal accelerates softening [9]. In addition, a lack of epidermal waxes was found to cause postharvest storage water loss in chili peppers [10], indicating that waxes are critical for fruit storage quality at the postharvest stage. However, this protective layer can be damaged or lost during harvesting, packaging, and transportation [11]. This appearance may also give the impression that the fruit is of poor quality.

The main cause of fruit softening is the breakdown of pectin, cellulose, and hemicellulose [12]. As the fruit softens, chelated pectin and soluble pectin are gradually converted to water-soluble pectin [13], and polygalacturonase (PG), pectin methylesterase (PME) and glycosidase (*β*-Gal) are the main enzymes that break down pectin substances. Reportedly, cellulose–hemicellulose degradation leads to changes in their network, thus ultimately resulting in fruit softening [14]. The three enzymes xyloglucan endotransglycosylases (XET), *β*-glucosidase (*β*-Glu), and cellulase (CEL) are closely associated with cellulose–hemicellulose degradation. These enzymes are also associated with cellulose–hemicellulose degradation in strawberry [15], kiwifruit [16], apricot [17], and winter jujube [18], thus highlighting the role of cell wall-degrading enzymes in fruit softening. Reportedly, PG, PME, *α*-ARF, and *β*-GAL activities were substantially higher in cantaloupe with broken epidermal wax compared to the control group, and its quality was reduced with softened texture [19]. However, little is known about the effects of epidermal wax loss on prune softening.

Fruit frosting is extremely easy to remove and often overlooked; however, it can be a key factor in accelerating the softening of prunes. Currently, there is limited research on the metabolic aspects of fruit frosting on prune cell walls. Therefore, in this study, ‘France’ prunes were selected and classified by the degree of frosting coverage on the fruit surface. Also, the quality indexes, cell wall-degrading enzyme activities, and cell wall microstructures of prune fruits were measured by sampling every 8 days after 56 days of storage at 1–2 °C. It is important to study the effect of the degree of frost coverage on postharvest quality changes and cell wall-degrading enzyme activities in prune fruits in order to reduce fruit losses during long-distance transport and as a theoretical basis for developing new methods of fruit and vegetable storage and preservation in the future.

## 2. Materials and Methods

### 2.1. Experimental Treatments and Plant Materials

‘France’ prunes were picked from the Kashgar prune orchard (75°51′42.786″ E, 39°22′32.887″ N) based on the criteria of uniform maturity, zero mechanical damage, firmness of 19.5–20 N, total soluble solids (TSS) of 23–24%, and uniform appearance, color, and size. To minimize fruit vibrational damage, immediately after harvest, they were transported by air to the storage and preservation laboratory of the Xinjiang Academy of Agricultural Sciences (Urumqi, China).

The simulation of the degree of frost coverage loss during picking and transport was divided into three levels, expressed in terms of the index of frost coverage (level 0: frost-less fruit; level 1: fruit frost covering 1/3–1/2 of the whole fruit; level 2: fruit frost area of 4/5–1). Furthermore, they were divided into three groups: the heavy fruit frosting group (HFF) comprising level 2 fruits; the light fruit frosting group (LFF) comprising level 1 fruits; and the frost-less fruit group (FF) comprising level 0 fruits. Fruit frost on the surface of the FF group was wiped off with a flannel cloth.

This is fruit frost cover index formula:

(1) $$\mathrm{Fruit}\;\mathrm{frost}\;\mathrm{cover}\;\mathrm{index}\;\mathrm{formula}=\frac{⅀\left(Fruit\;Frosting\;\;Levels\times Number\;of\;fruits\;at\;that\;level\right)}{Fruit\;Frosting\;\;Maximum\times Total\;number\;of\;fruit\;surveyed}$$

For each group, 10 kg of prune fruits were placed in plastic baskets of uniform size, which were packed using non-woven fabrics and fixed to a homemade tabletop shaker (Y90LL-2, Shenyang Hongwei Electric Machine Manufacturing Co., Ltd., Shenyang, China). The vibration data were based on the vibration frequencies measured by a truck used for the transportation of pears and plums [20]. The following parameters were used: frequency at 5 Hz, experiment duration of 8 h, treatment temperature set at 4 °C, relative humidity of approximately 95%, and vibrations packed into 0.02 mm PE film plastic bags. The samples were stored at 1–2 °C and were sampled once every 8 d. Each time, 15 random samples were selected for determining the quality indexes of prunes, and 15 random frozen samples were used for determining the subsequent related physiological indexes. All of the experiments were conducted with three replications.

### 2.2. Determination of Physiological Characteristics

Prune color was determined using a precision colorimeter (SC-10; Suzhou Xinmeihe Instrument Co., Ltd., Suzhou, China) for surface color (L*, a*, and b*), with 15 randomly selected samples from each treatment. Subskin color was determined on both sides of the equator thrice. The fruit hardness was determined separately for each treatment using a fruit firmness tester (GY-4, Yueqing Aidelberg Instrument Co., Ltd., Yueqing, China), and subskin hardness was determined twice at the equator for each fruit. The titratable acid (TA) content was determined using the method described by Ding et al. [21]. With minor modifications, 10 g of the fruit was ground, mixed with distilled water, and poured into a 100 mL volumetric flask for volume determination. After 30 min, the mixture was filtered, and 5 mL of the filtrate was added to the flask. Next, two drops of 2% phenolphthalein reagent were added, titrated with NaOH, and calculated according to the given formula. The total soluble solids (TSS) content of the prunes was determined using a PAL-1 digital saccharimeter, and the weight loss rate was determined according to the method described by Zhang et al. [8]. This was performed by weighing 1 kg of prunes and placing them in the same storage conditions, collecting samples every 8 d, weighing the mass of the fruits at each sampling time using an electronic balance, and calculating the rate of weight loss per unit of time, with the results expressed in % of cell membrane permeability. Based on the method described by Cui et al. [22], relative conductivity was used to indicate cell membrane permeability and the extent to which cell membranes were damaged. Relative conductivity is the percentage of the conductivity of the live tissue extracts of the test fruit to the conductivity of the boiled fruit tissue extract. All measurements were repeated three times, and the results were averaged.

### 2.3. Determination of Cell Wall Polysaccharide Content

The extraction of cell wall material (CWM) was conducted using a slight modification of the method described by Fan et al. [23]. From each of the three groups of frozen prune fruit samples, 25 g of fruit sample was selected, and 100 mL of pre-cooled 80% ethanol was added to thoroughly pulverize and mix the pulverized mixture, which was then placed into boiling water and refluxed for 1 h. After the above steps were repeated three times, the filtration residue was washed with a mixture of chloroform and methanol (1:1) thrice and was washed, filtered, and re-washed with acetone three times until the color of the insoluble substances disappeared. The color of the insoluble substance disappeared, the residue was collected by filtration and dried under vacuum at 45 °C until constant weight, and the obtained substance was CWM. The isolation of alcohol-insoluble cell wall polysaccharides, chelate-soluble pectin (WSP), chelate-soluble pectin (CSP), Na_2_CO_3_-soluble pectin (NSP), and cellulose from the cell wall material was continued according to the method described by Fan et al. [24]. A weight of 250 mg of cell wall was soaked in ultrapure water and then centrifuged at 12,000× *g* at 4 °C for 15 min after 6 h of standing. The supernatant was collected as WSP, soaked in 50 mmol/L pH 6.5 sodium acetate buffer (containing 50 mmol/L cyclohexane-trans-1 and 2- diamine tetra-acetate (CDTA)), and then centrifuged at 12,000× *g*, 4 °C for 15 min after 6 h of standing. Next, the obtained supernatant was collected as CSP and soaked in 50 mmol/L sodium acetate buffer (containing 50 mmol/L CDTA), and the supernatant was collected as CSP; and soaked in 50 mmol/L CDTA, and the supernatant was collected as CSP. The supernatant was allowed to stand for 6 h, centrifuged at 4 °C and 12,000× *g* for 15 min, and soaked in 50 mM sodium carbonate (containing 2 mM CDTA). WSP, CSP, and NSP were determined using carbazole colorimetry [25] and were expressed in units of g/kg. The cellulose content was determined according to the method of Chen et al. [26] and was expressed in units of g/kg.

### 2.4. Determination of Enzyme Activities Related to Cell Wall Degradation

Solutions of cell wall-degrading enzymes were extracted according to the methods described by Wang et al. [27]. Randomly selected 3 g of frozen prune samples were weighed and mixed into 6 mL of 95% pre-cooled ethanol, crushed and homogenized in an ice bath, transferred to a centrifuge tube and incubated for 10 min at 4 °C, and then centrifuged for 10 min at 12,000× *g* at 4 °C. After the supernatant was decanted, 3 mL precooled 80% ethanol was added to the residual precipitate, and then 5 mL precooled 50 mmol/L, pH 5.5 sodium acetate buffer (1.8 mol/L NaCl) was placed at 4 °C for 20 min and then centrifuged at 12,000× *g*, 4 °C for 20 min, and the supernatant was collected. Using the method of Zhao et al. [18], the activity of TPolygalacturonase (PG), the activity of cellulase (CEL), the activity of *β*-glucosidase (*β*-Glu), and the activity of *β*-galactosidase (*β*-Gal) were measured.

The activity of PME was determined as described by Liu et al. [28]. Four grams of the sample was ground with 8.8% (*w*/*v*) NaCl solution, shaken for 4 h, and centrifuged for 30 min. The supernatant was used as the PME extract. Next, 0.1 mL of the enzyme extract was added to a mixture containing 0.75 mL of water, 2.5% pectin 2.0 mL, and 0.01% bromothymol blue solution 0.15 mL and was read at a wavelength of 620 nm (T6 New Century, Beijing Puxi General Instrument Co., Ltd., Beijing, China).

Five grams of fruit jelly samples were weighed and homogenized in sodium acetate buffer (pH 5.2, containing 2% *β*-mercaptoethanol, 100 μmol/L NaCl, and 5% PVP) using the method of Zhao et al. [18]. Subsequently, the samples were centrifuged at 4 °C for 20 min, 15,000× *g*, and supernatants were extracted. Approximately 1.3 mL sodium acetate buffer (0.1 mmol/L, pH 5.0) and 0.2 mL 0.1% xylan were added to 0.5 mL of the supernatant, and the reaction was conducted for 1 h at 37 °C. The supernatant was then mixed with the DNS solution (1.5 mL), placed in a boiling water bath for 5 min, and cooled to determine the absorbance at 540 nm. The amount of enzyme required for the production of 1 mg of xylose per unit time was considered as one unit of enzyme activity (U).

### 2.5. Determination of Relative Gene Expression

Total RNA from prunes was extracted and reverse transcribed, and Primer Premier 6.0 and Beacon Designer 7.8 were used to design and synthesize the primers (Table 1). The relative expression of each gene was calculated using the 2^−ΔΔCt^ method with *GAPDH* as an internal standard. The real-time PCR reaction conditions were as follows: 95 °C, 1 min; 95 °C, 15 s; 63 °C, 25 s; 40 cycles. All qRT-PCR experiments were set up with three biological replicates, and three parallel determinations were made with reference to Ma et al. [13].

### 2.6. Microscopic Observations

Prunes were cut into 1 cm^3^ pieces, fixed in 2.5% (*v*/*v*) glutaraldehyde solution for 5 h at 4 °C, and washed with phosphate buffer (0.1 M, pH 7.0) for 15 min according to the method described by Zhao et al. [18]. The specimens were then fixed in 1% osmium acid (OsO_4_) solution for 2 h. Finally, the specimens were washed with phosphate buffer. The rinsed samples were dehydrated with a series of graded ethanol (30–95%) solution and acetone, treated with an embedding agent and a mixture of acetone, and heated at 70 °C for 9 h. The samples were sliced using LEICA EMUC7 (Leica Microsystems GmbH, Wetzlar, Germany), stained with uranium acetate and alkaline lead citrate for 5–10 min, and observed under a transmission electron microscope (Hitachi H-7650, Suzhou Sainz Instruments Co., Ltd., Suzhou, China).

### 2.7. Statistical Analysis

Calculations were performed using Excel and were plotted using Origin 2024 (SPSS 23.0, IBM Corp., Armonk, NY, USA), and significant differences were indicated at *p* < 0.05.

## 3. Results

### 3.1. Changes in Quality Parameters of Prunes during Storage

**Figure 1:**
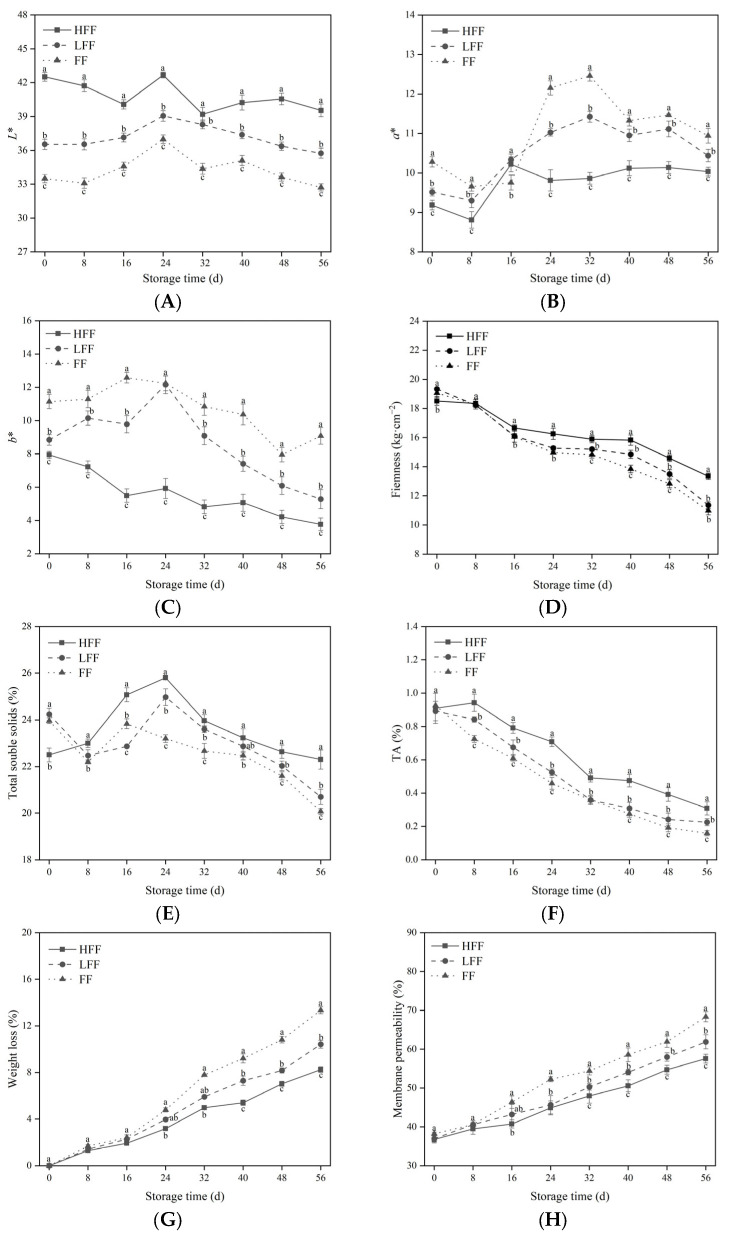
Changes in fruit color (L*, a*, b*) (**A**–**C**), firmness (**D**), TSS (**E**), TA (**F**), weight loss (**G**), and cell membrane permeability (**H**) during storage in three groups with different degrees of frost. The vertical line represents the standard deviation of the mean. Within the same time period, different letters indicate statistical significance between groups *p* < 0.05.

The organoleptic quality of a fruit is one of the criteria used to judge its quality [29]. As shown in Figure 1, the prune L* values (Figure 1A) decreased in the FF group, but the a* (Figure 1B) and b* values (Figure 1C) were significantly higher (*p* < 0.05). Throughout the storage period, the firmness of all three fruit groups showed a decreasing trend, and the firmness of the prunes in the HFF group was higher than that of the FF group. On day 56, fruit firmness in the HFF group was 17.5%, which was 21.5% higher than in the LFF and FF groups, respectively (*p* < 0.05) (Figure 1D).

TA and TSS are important indicators of fruit flavor. Notably, the TA content (Figure 1E) and TSS content (Figure 1F) were higher in the HFF group than in the FF group, which effectively maintained the quality of the pruned fruit. With the extension of the storage time, the weight loss rates of all three groups increased (Figure 1G). The weight loss rate of FF group was the largest, and the weight loss rate of HFF and LFF groups increased slower; at the end of the storage period, the weight loss rate of HFF and LFF groups decreased by 38.16% and 21.89%, respectively, compared with that of FF group, and the weight loss rate of the HFF group was 20.83% lower than that of LFF (*p* < 0.05), which indicated that the fruit frosting could significantly inhibit the postharvest water loss. The cell membrane permeability of the prunes tended to increase during the entire storage period (Figure 1H); however, it was still lower in the HFF group than in the other two groups, and a significant difference was observed at day 40 (*p* < 0.05). The HFF group had higher permeability than the other two groups, and the HFF group had lower permeability than the other two groups. Fruit frosting significantly affected the cell membrane permeability of prunes, which was reduced by 15.77%, 6.97% in the HFF group compared to the LFF groups and FF groups (*p* < 0.05).

### 3.2. Changes in WSP, CSP, NSP, and Cellulose Content during Fruit Storage

**Figure 2:**
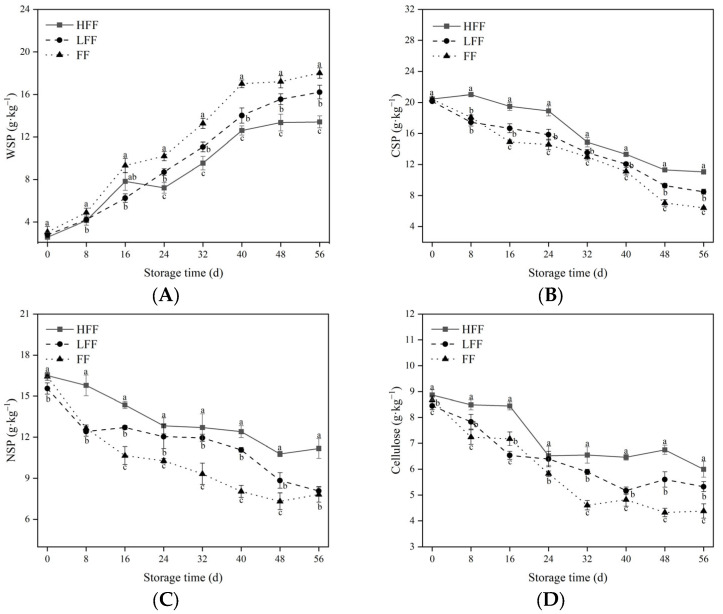
Indicates changes in the cell wall polysaccharide content of prune fruit, WSP (**A**), CSP (**B**), NSP (**C**), and cellulose content (**D**). The vertical line represents the standard deviation of the mean. Within the same time period, different letters indicate statistical significance between groups; *p* < 0.05.

The content of cell wall polysaccharides can indirectly reflect the shelf life and food value of fruits. Pectin and cellulose are important components of the fruit cell wall. Pectin within the fruit can be divided into WSP, CSP, and NSP. Throughout the storage period, the WSP content increased in all three groups, but the CSP and NSP contents decreased (*p* < 0.05). The HFF group showed the lowest WSP values (Figure 2A) and highest CSP (Figure 2B) and NSP (Figure 2C) contents. On day 56, the WSP content of the fruits in the HFF group was 17.29% and 25.50% lower than that in the LFF and FF groups, respectively, whereas the CSP content was 30.02% and 72.11% higher, and the NSP content was 38.41% and 42.83% higher (*p* < 0.05). As shown in Figure 2D, the cellulose content in the HFF group was consistently higher than that in the other two groups, and the cellulose content in the HFF group increased by 36.89% at the end of the storage period (*p* < 0.05).

### 3.3. Enzyme Activity during Cell Wall Changes

**Figure 3:**
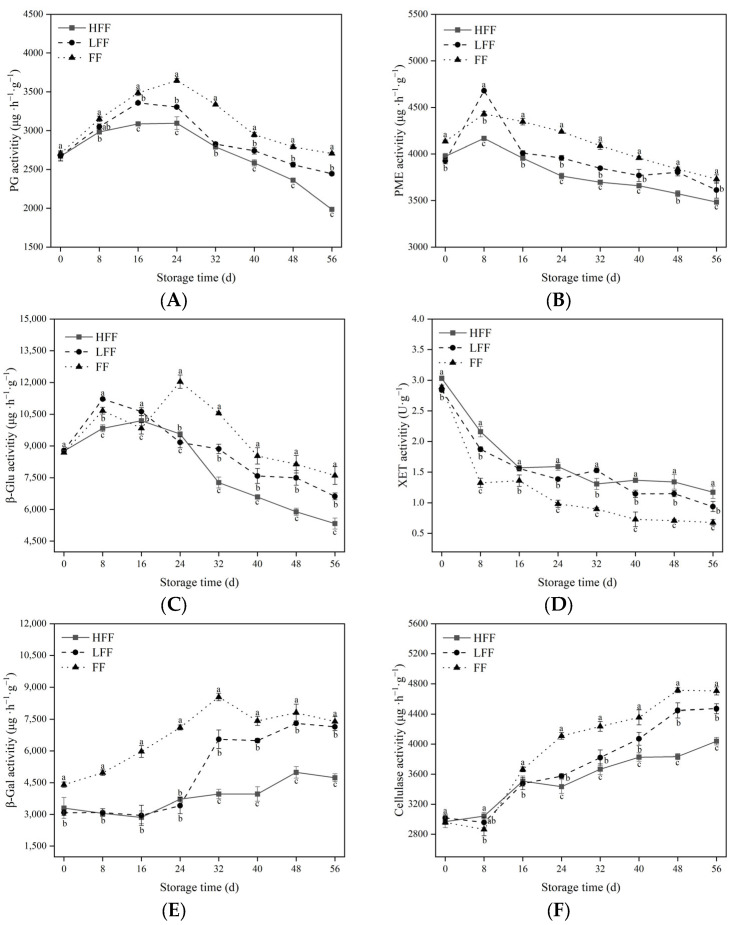
Changes in cell wall-degrading enzyme activity during storage: PG (**A**), PME (**B**), *β*-Glu (**C**), XET (**D**), *β*-GAL (**E**), CEL (**F**). The vertical line represents the standard deviation of the mean. Within the same time period, different letters indicate statistical significance between groups; *p* < 0.05.

In this experiment, the activity of PG (Figure 3A), PME (Figure 3B), and *β*-Glu (Figure 3C) in prunes of all three groups showed a tendency of increasing and then decreasing; after 24 d of storage, the measured factors of fruits in the LFF and FF groups were consistently higher than the fruits of the HFF group. At the end of the storage period, the PG, PME, and *β*-Glu activities of fruits in the FF group were increased by 36.32%, 7.10%, and 42.53% (*p* < 0.05) compared to those of the HFF group. Therefore, the lack of fruit frost coverage on the fruit surface increased the activity of PG, PME, and *β*-Glu, and the degradation of pectin under vibration stress could be inhibited. In contrast, the activities of *β*-Gal (Figure 3E) and CEL (Figure 3F) showed an elevated trend, and after 56 d of storage, the contents of *β*-Gal and CEL in the fruits of the HFF group decreased by 14.16% and 35.98%, respectively, compared with those of the FF group (*p* < 0.05). However, the XET activity in the HFF group showed a decreasing trend throughout the storage process, and the LFF and FF contents were consistently higher (Figure 3D). At the end of the storage period, CEL activity was reduced by 14.16% (*p* < 0.05) in the HFF group compared to that in the FF group.

### 3.4. Effect on the Expression of Genes Related to Fruit Softening

**Figure 4:**
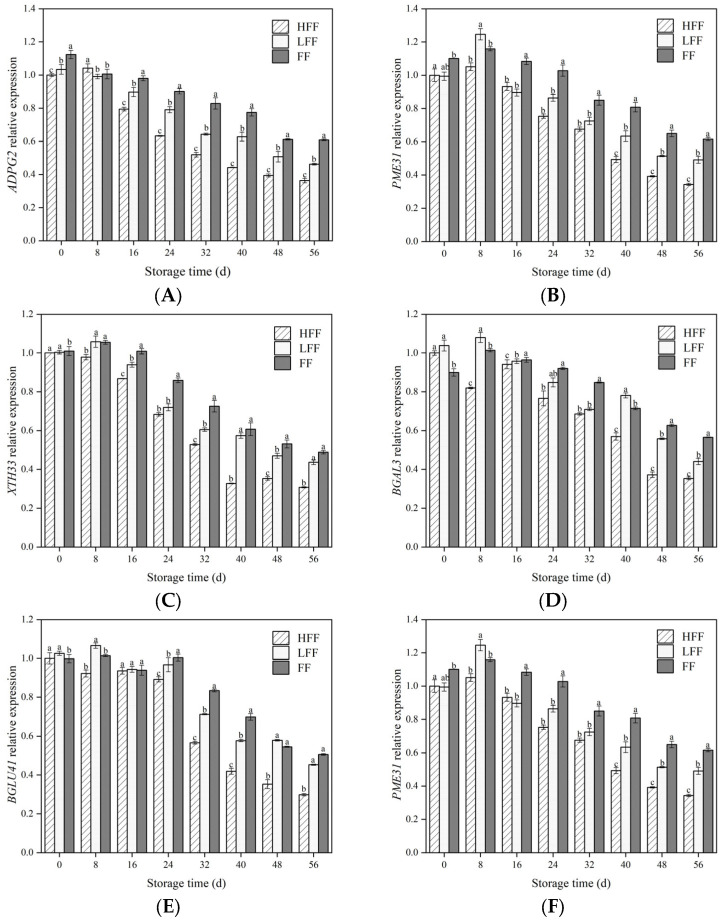
Changes in cell wall-degrading enzyme-related genes during storage: *ADPG2* (**A**), *PME31* (**B**), *XTH33* (**C**), *BGAL3* (**D**), *BGLU41* (**E**), and *CESA1* (**F**). The vertical line represents the standard deviation of the mean. Within the same time period, different letters indicate statistical significance between groups; *p* < 0.05.

The expression of *ADPG2*, *PME31*, *CESA1*, *BGAL3*, *XTH33*, and *BGLU41* was also investigated. As shown in Figure 4, *ADPG2*, *PME31*, *CESA1*, *BGAL3*, *XTH33*, and *BGLU41* showed an increasing–decreasing trend in all experimental groups during storage (*p* < 0.05), and gene expression was lower in the LFF and FF groups than in the FF group (Figure 4A–F). At the end of storage, the gene expression levels of *ADPG2*, *PME31*, *CESA1*, *BGAL3*, *XTH33*, and *BGLU41* in the HFF group were 40.39%, 44.32%, 41.06%, 37.52%, 37.22%, and 41.11% lower, respectively, than those in the FF group (*p* < 0.05). In summary, we speculate that fruit frosting may inhibit the upregulation of genes encoding cell wall-degrading enzymes.

### 3.5. Microscopic Observations

**Figure 5:**
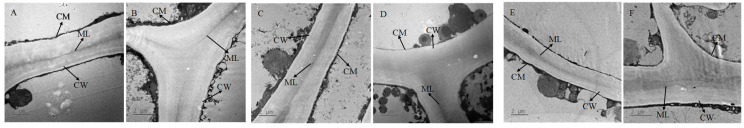
Transmission electron microscopy (TEM) images at 0 d after the end of vibration. ML: middle lamella CW: cell wall CM: cell membrane. HFF group (**A**,**B**), LFF group (**C**,**D**), FF group (**E**,**F**).

**Figure 6:**
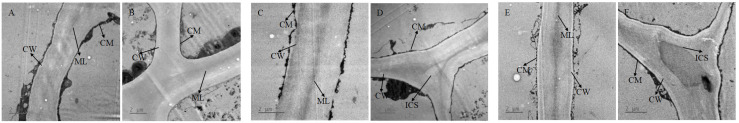
Ultrastructure of prune tissue on day 24 of storage. ML: middle lamella. CW: cell wall. CM: cell membrane. ICS: cell gap. HFF group (**A**,**B**); LFF group (**C**,**D**); FF group (**E**,**F**).

**Figure 7:**
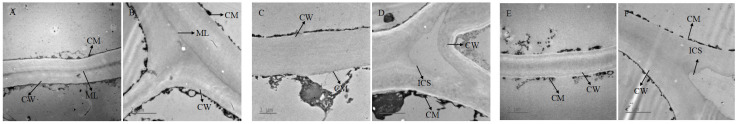
Ultrastructure of prune tissue on day 56 of storage. ML: middle lamella. CW: cell wall. CM: cell membrane. ICS: cell gap. HFF group (**A**,**B**); LFF group (**C**,**D**); FF group (**E**,**F**).

As shown in Figure 5, on day 0, the cell wall structure of pruned fruits in the HFF and LFF groups remained intact. The cell wall was closely related to the cell membrane, forming a light–dark–light-region structure; the triangular structure was complete, and the middle lamella was obvious (Figure 5A–D). In contrast, fruit cell walls in the FF group were curved (Figure 5E,F), and the HFF group had the highest fruit hardness. On the 24th day after harvest, the gelatinous layer in HFF fruits was still clearly visible, and the structure of the delta area was relatively complete (Figure 6A,B). At this point, the cell gap of the FF group fruits increased, and the cell wall of the delta area appeared to be flocculent by bending and fracturing the cell wall. Twenty-four days after harvest and storage, the cells of the FF group had already appeared to soften, and the cell wall was bent and deformed (Figure 6C,D), whereas the fruits in the HFF group maintained a better cellular structure and delayed the occurrence of fruit softening. The onset of fruit softening is depicted in Figure 6E,F. After 56 d of storage, all three groups underwent different degrees of changes. The cell wall of prune fruits in the FF group was obviously deformed (Figure 7E,F); the cell membrane structure was basically destroyed, was folded, and began to degrade or even disappeared, while the middle layer gradually dissolved, the thickness of the triangle was not uniform, and the cell gap was obviously enlarged. The cell wall of fruits in HFF group was slightly deformed (Figure 7A,B), but the cell membrane appeared to be degraded, and the structure of the triangle was relatively complete, but cell gap appeared. The structure of the triangle was relatively intact; however, cell gaps appeared. Notably, the fruit frost had a great protective effect on the cell wall structure of prune fruits.

## 4. Discussion

The epidermal wax of fruit is a protective layer that plays an important role in fruit growth and storage [30]. In the present study, the absence of frost on fruit affected the original color of the prunes. A decrease in L* produces a deepening of the fruit color, darker fruits, and a decrease in the intensity of the blue color, leading to an increase in the a* and b* values. This gives the impression that the fruit is of poor quality. Firmness reflects the degree of softening of fruit quality during storage. During storage, the firmness of fruits without fruit frosting was significantly lower than that of the heavy fruit frosting group, which accelerated the decline in firmness. These results indicate that fruit frosting contributes to maintain fruit firmness and delays fruit softening. In addition, fruit sugars and acids determine the flavor of the fruit to some extent. During postharvest storage of the fruit, organic acids are gradually decomposed, accompanied by the accumulation of sugars [31]. In the present study, the TSS content of the HFF group was consistently higher than that of the FF group, indicating that the fruit frosting could retard the range of responses of prune fruits and reduce the consumption of nutrients, reducing the original flavor of the fruit. Similarly, the TA content of the HFF group was high, indicating that fruit frosting was beneficial for maintaining the texture and flavor of prune fruits. The rate of weight loss is inextricably linked to the moisture content of the fruits during storage. In this study, frost-less fruits experienced accelerated rates of weight loss, whereas the opposite was true for fruits in the HFF group. Thus, it was shown that the frosting on the surface of prunes effectively maintains the firmness of the fruit and prevents water loss due to transpiration, which is consistent with the findings of Zhang et al. [8] on cantaloupe.

Cell walls play an important role in the plant body, and they consist of polysaccharide fractions (pectin, cellulose, and hemicellulose). The degradation of pectin (WSP, CSP, and NSP) leads to cellulose and hemicellulose solubilization, reduced cell adhesion, and ultimately softening of the fruit [32,33]. In this study, the FF group showed a rapid decrease in CSP, NSP, and cellulose content throughout the study period when compared to the HFF group, but the FF group showed an upward trend in WSP. This shows that CSP and NSP are continuously converted to WSP during storage, leading to loosening and degradation of the cell wall structure and gradual softening of the fruit [18]. Fruit frosting can effectively delay the degradation of cell wall polysaccharide components, inhibit fruit softening, and maintain fruit firmness.

Fruit firmness is closely related to the activities of cell wall-modifying enzymes such as PG, PME, CEL, *β*-GAL, *β*-Glu, and XET [34,35]. The degradation and transformation of pectin substances are mainly related to PME and PG, which together degrade pectin substances, destroy the cell wall mesostructure, and decrease cell-wall cohesion during fruit ripening [36]. The products following the action of PME are further hydrolyzed by PG, which catalyzes the cleavage of the pectin molecule, the final step being carried out by *β*-Gal, which hydrolyzes the sugar chain, glycolipids, and the non-reducing terminal *β*-D-galactose residues of glycoproteins, leading to an increase in the content of WSP and, ultimately, to the degradation of pectin [37]. *β*-Glu can hydrolyze the *β*-1,4-glycosidic bond and release glucose from the non-reducing end. It is also a kind of cellulase and is the rate-limiting enzyme in the process of cellulose hydrolysis [38]. In addition, PME, PG, and CEL work together to convert cellulose into glucose, leading to cell wall loosening and fruit softening [35]. In this experiment, heavy fruit frosting inhibited the increase of PG, PME, CEL, *β*-Glu, and *β*-Gal activities, which was beneficial to maintaining the pectin content and cellulose content of the fruits. It is possible that the surface of the fruits was protected by the fruit frosting, which prevented the loss of water from the interior of the fruits. Similar results were obtained for the removal of cantaloupe cuticle waxes [19]. However, we found that XET activity showed a decreasing trend in this study and was found to be significantly higher in the HFF group than in the other two groups. XET acts as a catalyst for the transfer and hydrolysis of xyloglucan molecules and alters the xyloglucan–microfibrillar filament network structure, leading to cell wall relaxation and expansion [39]. It may participate in the process of assembling new components of the cell wall, resulting in its structural strengthening of the cell wall. Castro et al. [40] found that XET is also associated with cell wall synthesis; however, its mechanism of action needs to be further explored.

Cell wall-degrading enzymes work together to promote decreased fruit firmness, pectinolysis, and cell wall structural relaxation [41]. However, these cell wall-degrading enzyme activities are affected by the expression of degrading enzyme genes, and the expression levels of *ADPG2*, *PME31*, *CESA1*, *BGAL3*, *XTH33*, and *BGLU41* are strongly correlated with the activities of PG, PME, CEL *β*-Glu, and *β*-Gal, respectively, which ultimately lead to the solubilization of pectin and cellulose [23,42]. In this study, we have analyzed the expression of genes related to cell wall-degrading enzymes and found that the expression of each enzyme gene was down-regulated in the HFF group and inhibited the increase of PG, PME, *β*-Glu, *β*-Gal, and CEL activities. Also, we found that the cell wall polysaccharides had a higher content, which led to maintaining a higher firmness and delayed the softening of the fruits. Thus, it is suggested that these genes are able to regulate the activity of the corresponding enzymes in the fruit during fruit frosting, which in turn affects the subsequent degradation of cell wall polysaccharides, which was similar to the results in apricot [43] and lychee [28].

To better understand the changes in the internal cell wall structure of the fruit, transmission electron microscopy was used in order to observe the ultrastructure of the cell wall of the fruit during the storage period. This showed that the cell wall structure of the FF group gradually relaxed, and the dissolution of the mesocarp layer led to an increase in the cell gap, which affected the integrity of the cells. In contrast, the other two groups of fruits showed delayed cell wall loosening and decomposition and maintained their structural integrity. This suggests that fruit frosting can effectively inhibit the decomposition of cell wall structures and maintain fruit quality during storage.

In summary, heavy fruit frosting significantly inhibited PG, PME, *β*-Gal, and CEL viability; delayed the decline of NSP, CSP, and cellulose content; and inhibited the increase of WSP and cell membrane permeability when compared with the FF group. Observations of the cellular ultrastructure revealed that heavy fruit frosting could reduce the degradation of the internal structure and cell wall of apricot fruit cells, maintain cellular integrity better, and delay the decline in firmness compared with the control group. Therefore, fruit frosting is biologically important for maintaining the postharvest quality of prune fruits and delaying softening, and it is recommended that the frosting on prunes should be preserved at harvest in order to prevent fruit quality decline. However, this study only analyzed the effect of fruit frosting on the postharvest storage quality of the prune fruits, and it remains to be clarified how the specific components of fruit frosting are related to fruit storage quality.

## 5. Conclusions

Changes in the firmness of prune fruits during storage are related to the degradation of pectin and cellulose caused by cell wall-degrading enzymes. The changes in cell wall-degrading enzyme activities in heavily frosted fruits were attributed to the fact that frosting helped to maintain fruit moisture; downregulated the expression levels of *ADPG2*, *PME31*, *CESA1*, *BGAL3*, *XTH33*, and *BGLU41*; slowed down the hydrolysis of polysaccharides; and maintained the cell wall structure of prune fruits, which in turn slowed down the softening of the fruits and prolonged the storage period.

## Figures and Tables

**Table 1:** Primer sequences.

Gene Name	Forward Primer	Reverse Primer
*GAPDH*	CCATGGGGAAGGTGAAGGTC	TCGCCCCACTTGATTTTGGA
*ADPG2*	AGTTGCACTGAGGCAGAACA	CACTAGCGCATCGGAAAACG
*PME31*	TGTCGATTTCTCGGTTGGCA	CAGTGTTCCAAGAGAGCGGT
*CESA1*	AACAGTGGCTACCAGTCGTG	ATTGGCGTCCACAAAGGGAT
*BGAL3*	TCGAGGAGCTTGGTGGAAAC	CTGCGTGACACTCTCCTTGT
*BGLU40*	ACGACGCCGTTTCTGATTCT	ATGCTGTTTGGGTCGTCCAT
*XTH33*	GTGACACGCCTCACAGATCA	AGAGTGAGCTTAGCGAGGGA

## Data Availability

The original contributions presented in the study are included in the article. Further inquiries can be directed to the corresponding author.
